# High-performance sport in a developing country through the lens of coaches and sports directors

**DOI:** 10.3389/fspor.2026.1877793

**Published:** 2026-09-03

**Authors:** Mauricio Garzon, Emilie Malcourant, Alain Steve Comtois, Tegwen Gadais, Mathieu Winand

**Affiliations:** 1 Département des Sciences de L'activité Physique, Université du Québec à Montréal, Montreal, QC, Canada; 2 Université Catholique de Louvain, Louvain-la-Neuve, Walloon Brabant, Belgium; 3 New International University for Sport and Health, LUNEX University, Differdange, Luxembourg, Luxembourg

**Keywords:** coaching development, factors affecting performance, high performance coach, perception of coaches, sport in developing countries

## Abstract

The importance of identifying the key factors for success in high performance sport has been studied for several years. Although the focus during the competition is on the sporting result, it is important to note that the result in sport is preceded by a series of circumstances and factors closely linked to the context of the athlete's preparation process. Therefore, this study aims to identify the potential factors affecting the high-performance sport in a developing country like Colombia from the perception of high-performance coaches. High-performance coaches (HPC) and high-performance directors (HPD) were asked to answer two questionnaires. Descriptive and factorial analyses were performed to identify the decisive factors. Likewise, a one-way ANOVA between groups was performed to assess whether the responses were significantly different. The principal results showed that more than half of the factors identified by developed countries as determinants of success in high performance sport are perceived as weaknesses within the current high-performance system of the emerging country studied. The factors that emerged from this study were Process, Technical and social support, Structure of sports policies, Competition level, and Contractual conditions. This study identifies some factors that have been limiting or facilitating the results of Colombian athletes in elite sports. It also opened new perspectives of research on the field of HPC and HPD, especially inside Latin America Caribbean region and for developing countries more generally.

## Introduction

Over the past five decades, the development of sport policy has been largely characterized by the dual objectives of promoting elite sport and expanding mass participation. Within this context, high-performance sport and international sporting success have increasingly become political priorities for many governments (1–5). Consequently, the success of National Olympic Committees (NOCs) and their affiliated National Federations (NFs) is commonly assessed through athletes' performances at major international competitions, including the Olympic Games, World Championships, and continental multi-sport events.

A growing body of research is focusing on the performance of National Governing Bodies (NGBs), and the determinants of international sporting success. However, this literature has predominantly focused on highly industrialized nations, where the interaction between social, political, economic, and cultural conditions has shaped the development of sophisticated elite sport systems (1, 6–10). Consequently, “if nations wish to maximize the likelihood of success at the Olympic Games, they must not only design and develop effective elite sport policies, but also have the right personnel in place to lead and manage their Olympic programs, creating the optimal environment, implementing systems and structures, developing an inclusive culture and providing appropriate support” (11).

These countries are further distinguished by substantial public investment in elite sport, the strategic allocation of financial and organizational resources, and the political and ideological significance attached to international sporting success (12–14). In contrast, in the context of developing countries, sports organizations face significant challenges in mobilizing resources for sports development at all levels, from grassroots to elite sport.

In recent years, sports management has focused its attention on the discrepancy between governance and sports policy, an issue that has gained prominence (15–17).

The competitive performance of Latin American countries at major international events illustrates these structural disparities. Cuba remains the only country in the region to have consistently ranked among the top five nations at the Olympic Games, reflecting the integration, at that time, of several key factors of elite athletic performance. Similarly, Brazil achieved its highest-ever Olympic ranking (13th place) when hosting the Rio 2016 Olympic Games, following substantial investments across multiple dimensions identified by the SPLISS framework as critical to elite sport success (18, 19).

In the case of Colombia, a substantial improvement in high-performance sports results was observed between 2000 and 2016, attributable to the implementation of strategies analogous to those employed in developed countries. These strategies encompassed the establishment of a framework for planning, technical-scientific monitoring, and financial support for athletes identified as potential medalists at both national and international levels (20–24).

However, these policies were not sustained over time due to significant organizational and governance limitations that hinder the development of a successful elite sports system. Consequently, since 2016, Colombia's international performance has generally declined, with notable exceptions, such as artistic gymnastics, as reflected in the results of the Tokyo 2020 and Paris 2024 Olympic Games. Such conditions challenge the resource-based view of competitive advantage, which emphasizes that organizational success depends not only on the availability of resources but also on the capacity to deploy them strategically and effectively (25, 26).

Effective governance of high-performance sport requires leadership capable of promoting and safeguarding the fundamental values underpinning sport, including health promotion, social inclusion, non-discrimination, fair play, transparent athlete management, integrity, and teamwork. These principles should guide the actions of coaches, managers, and all stakeholders involved in elite sport systems (27). Within this framework, the perspectives of elite athletes, coaches, and performance directors are essential for evaluating sport policies, identifying critical success factors, and informing strategic decisions aimed at enhancing international competitive performance.

For this reason, it is important to examine whether the factors associated with elite sporting success in developed countries also manifest in emerging sports systems such as Colombia's, even knowing the limitations related to the non-equivalence and reliability of the concepts and instruments used in these studies to measure and compare the same phenomena in different contexts (28).

One approach to enhancing the validity and reliability of context-specific data is the development of a conceptual framework that clearly defines the constructs underpinning survey instruments. Equally important is the establishment of rigorous inclusion criteria to ensure that respondents possess the expertise and experience necessary to provide informed judgments. In the present study, these criteria were applied to the selection of high-performance coaches and high-performance directors (see Methods section). Examining their perceptions offers valuable insights into evidence-informed decision-making and the future development of elite sport policy in developing countries.

Therefore, this study aims to identify the potential factors affecting the high-performance sport in a developing country like Colombia from the perspectives of high-performance coaches (HPCs) and high-performance directors (HPDs), thereby contributing to a better understanding of elite sport development within the context of an emerging sport system.

## Materials and methods

### Participants

This study employed a non-experimental, descriptive research design (29). Participants were selected using purposive sampling, an approach widely adopted in qualitative research to identify information-rich participants with substantial expertise and experience relevant to the phenomenon under investigation (11, 30). A total of 42 participants were recruited. All participants were informed of the study objectives and provided informed consent before completing the questionnaires.

To be eligible for inclusion, high-performance coaches (HPCs) were required to have coached at least one elite athlete competing in either Olympic or Paralympic sport. Consistent with the definition proposed by De Bosscher and De Rycke (2017), an elite athlete was defined as an able-bodied athlete who, individually or as part of a team, had achieved a top 16 ranking in senior world-level competitions or a top 12 ranking in an equivalent continental championship (18). Given the absence of a comparable definition for Paralympic sport, this study defined Paralympic elite athletes as those who had won at least one medal at the Parapan American Games, representing the highest level of continental competition preceding the Paralympic Games.

The final sample comprised 14 high-performance coaches from Olympic (conventional) sports (HPCc), 14 high-performance coaches from Paralympic sports (HPCp), and 14 high-performance directors (HPDs). The HPCs were responsible for coaching athletes preparing for the Tokyo 2020 Olympic and Paralympic Games. On the other hand, to be eligible, high-performance directors (HPDs) were required to have served as the director of a Departmental Sports Institute (DSI) or to have managed a high-performance program within a DSI at any time during the previous 20 years. This group also included representatives from the Colombian Olympic Committee and the Ministry of Sport.

### Data collection

The high-performance directors (HPDs) and high-performance coaches (HPCs) were initially contacted by telephone and email to inform them of the objectives of the study, the nature of their participation, and the questionnaires to be completed. Participants who agreed to participate received questionnaires, and a conceptual framework defining the principal constructs assessed in the questionnaires to ensure a common understanding of the terminology.

Participants were informed that any questions regarding the completion of the questionnaires would be addressed directly by the principal investigator. Both questionnaires also included opportunities for respondents to provide written comments for each item and to identify additional factors they considered important for achieving success at the highest levels of international competition (i.e., World Championships, Olympic Games, and World Games) that were not represented in the original instruments.

The questionnaires were developed from previous research examining elite coach development, support systems for high-performance coaching, coach education, employment conditions, and organizational factors influencing coaching effectiveness in high-performance sport (1, 6, 19, 31–33). Content validity was established through expert review by all members of the research team, after which the instruments were translated and culturally adapted into Spanish.

The first questionnaire, Support Mechanisms Available (SUMA) (Supplementary File 1), comprised 20 dichotomous (Yes/No) items designed to determine the extent to which the Colombian high-performance sport system provides organizational conditions that facilitate coaches' professional development and enable them to maximize their coaching potential. For each item, respondents indicated whether the corresponding support mechanism was present within the Colombian high-performance sport system. Positive responses (“Yes”) were coded as 1 and negative responses (“No”) as 0.

In order to provide a better understanding of the coach's development in developing countries (CDDC), a second questionnaire, Perception of Factors for Success (PERFS) (Supplementary File 2), consisted of 17 items assessing the perceived importance of factors contributing to athletes' success at the highest international competitive level. Respondents rated each factor on a 10-point Likert scale ranging from 1 (not at all important) to 10 (extremely important). Participants were instructed to evaluate each factor according to its practical importance in achieving elite performance based on their own professional experience rather than its theoretical relevance. All questionnaires were reviewed upon receipt, and respondents were contacted whenever clarification or completion of missing responses was required.

### Data analysis

Responses from both questionnaires were operationalized as quantitative variables to facilitate statistical analysis. Given that the study focused on a highly specialized population of high-performance directors (HPDs) and high-performance coaches (HPCs), selected according to pre-established criteria, who represent a substantial or complete proportion of the population of high-performance sports coaches and managers in Colombia, factor analysis should be interpreted as an exploratory data reduction technique aimed at identifying preliminary patterns amongst the variables. Exploratory factor analysis can recover meaningful factor structures with sample sizes below 50 when factor loadings are high and the number of variables per factor is sufficient (34, 35).

For the SUMA questionnaire, affirmative responses were assigned a value of one (1) and negative responses a value of zero (0). For each item, the number of affirmative responses was divided by the total number of participants within each group (HPD, HPCc, and HPCp) to calculate the percentage of respondents indicating that the support mechanism was available. Descriptive statistics (mean ± standard deviation) were calculated to characterize the coaching environment within the Colombian high-performance sport system.

An overall mean score representing the average number of affirmative responses was calculated for each participant group. Differences among groups were examined using one-way analysis of variance (ANOVA). When significant group differences were identified, Holm–Bonferroni *post hoc* comparisons were performed. To facilitate interpretation and support policy recommendations, responses for each SUMA item were classified into five categories according to the percentage of affirmative responses: Weakest (≤20%), Weak (>20% to ≤40%), Average (>40% to ≤60%), Strong (>60% to ≤80%), and Strongest (>80%).

For the PERFS questionnaire, descriptive statistics (mean ± standard deviation) were calculated for each item. Exploratory factor analysis was subsequently conducted to identify the underlying dimensions perceived by HPDs and HPCs as contributing to successful elite sport performance. Factors with eigenvalues ≥1.0 were retained following Kaiser's criterion. Varimax rotation was applied to improve factor interpretability, and factor loadings ≥0.50 were considered meaningful for factor retention.

Differences in PERFS scores among HPDs, HPCc, and HPCp were examined using one-way ANOVA. Where statistically significant differences were identified, Fisher's Least Significant Difference (LSD) *post hoc* test was performed for pairwise comparisons. Statistical significance was established at *p* < 0.05. All analyses were conducted using IBM SPSS Statistics version 21.

## Results

The descriptive analysis of the 20 items included in the Support Mechanisms Available (SUMA) questionnaire yielded an overall mean score of 9.14 ± 2.98 positive responses (out of a maximum of 20). When analyzed by participant group, the mean scores were 9.64 ± 2.90 for high-performance directors (HPDs), 8.43 ± 3.90 for high-performance coaches in Olympic sports (HPCc), and 9.36 ± 1.78 for high-performance coaches in Paralympic sports (HPCp). These results indicate that, on average, fewer than half of the support mechanisms identified in the literature as characteristic of successful elite sport systems were perceived to be present within the Colombian high-performance sport system. No statistically significant differences were observed among the three participant groups [*F_(2,39)_* *=* *0.622, p* *=* *0.542*].

Table 1 presents the percentage of affirmative responses for each item of the SUMA questionnaire, ranked from the lowest to the highest level of perceived availability. Item 16, *Elite coaches receive a post career support program to prepare and assist for life after sport,* received no affirmative responses, indicating that none of the participants perceived this support mechanism to be available. Conversely, Item 13, *Coaches have a written work contract for training activities; the job of a coach is contractually recognized and protected*, received 41 affirmative responses out of a possible 42, representing the highest level of agreement among respondents.

**Table 1:** Positive responses on the evaluation of the context of coach development in developing countries (CDDC).

Question	Question	YES	%	Level	
16	Elite coach post career support.	0	0	1 (≤ 20%)	Weakest
4	Coordinating agency responsible for coaches' education.	3	7		
5	The organization aligns with the different levels of NGB courses.	3	7		
15	Trade union for sports coaches and trainers.	4	10		
6	Coach education system.	7	17		
1	Database of coaches and elite coaches.	12	29	2 (≤ 40%)	Weak
18	Coaches working with talent development are paid.	13	31		
3	Strategy to attract the world's best coaches.	14	33		
12	The job of a coach is recognised in the country.	15	36		
17	A coaching qualification is mandatory to work.	15	36		
2	Coaches have experience as an athlete.	22	52	3 (≤ 60%)	Average
7	Continuous professional development of coaches.	22	52		
9	Coaches' general monthly income is sufficiently.	23	55		
11	Employers considering the training needs of elite coaches.	23	55		
20	Coaches are allowed to earn additional money.	23	55		
14	Contract is renewed annually.	30	71	4 (≤80)	Strong
19	Coaches earn bonuses ($) related to the performance.	35	83	5 (>80)	Strongest
10	Elite sport coaching is a full-time primary activity.	39	93		
8	Coaches receive specialist advice from other areas.	40	95		
13	Coaches have a written work contract for training activities.	41	98		

Each question is summarized by a short sentence. Each complete question can be found in Supplementary File 1.

**Figure 1:**
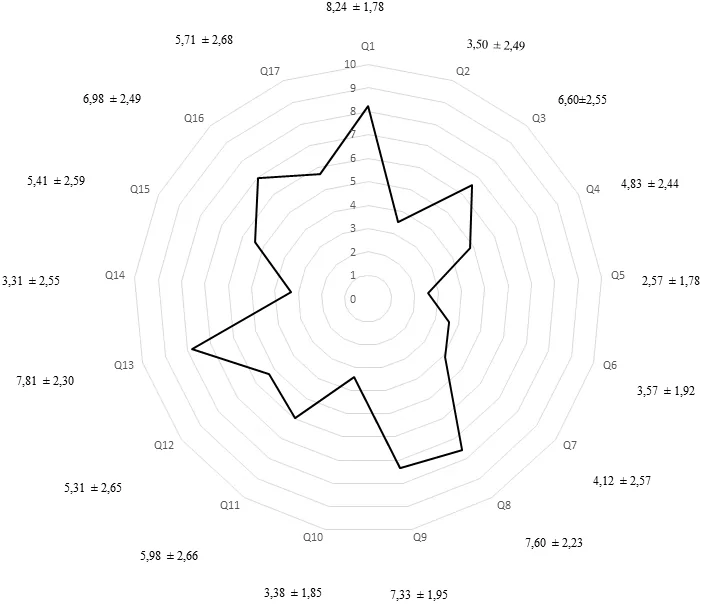
Radar graph by average perception for both HPD and HPC of the priority aspects to have achieved the results in Pan-American Games, World Championships, and Olympic Games. Mean values ± SD are showed for questions (Q 1 to Q17).

Responses to the Perception of Factors for Success (PERFS) questionnaire are summarized in Figure 1 using mean scores (± SD) for all participants. Based on the 10-point Likert scale, *Financial support from the government* (Item 1) received the highest importance rating, whereas *The appropriate policies in physical education* (Item 5) received the lowest mean rating among the factors evaluated.

**Figure 2:**
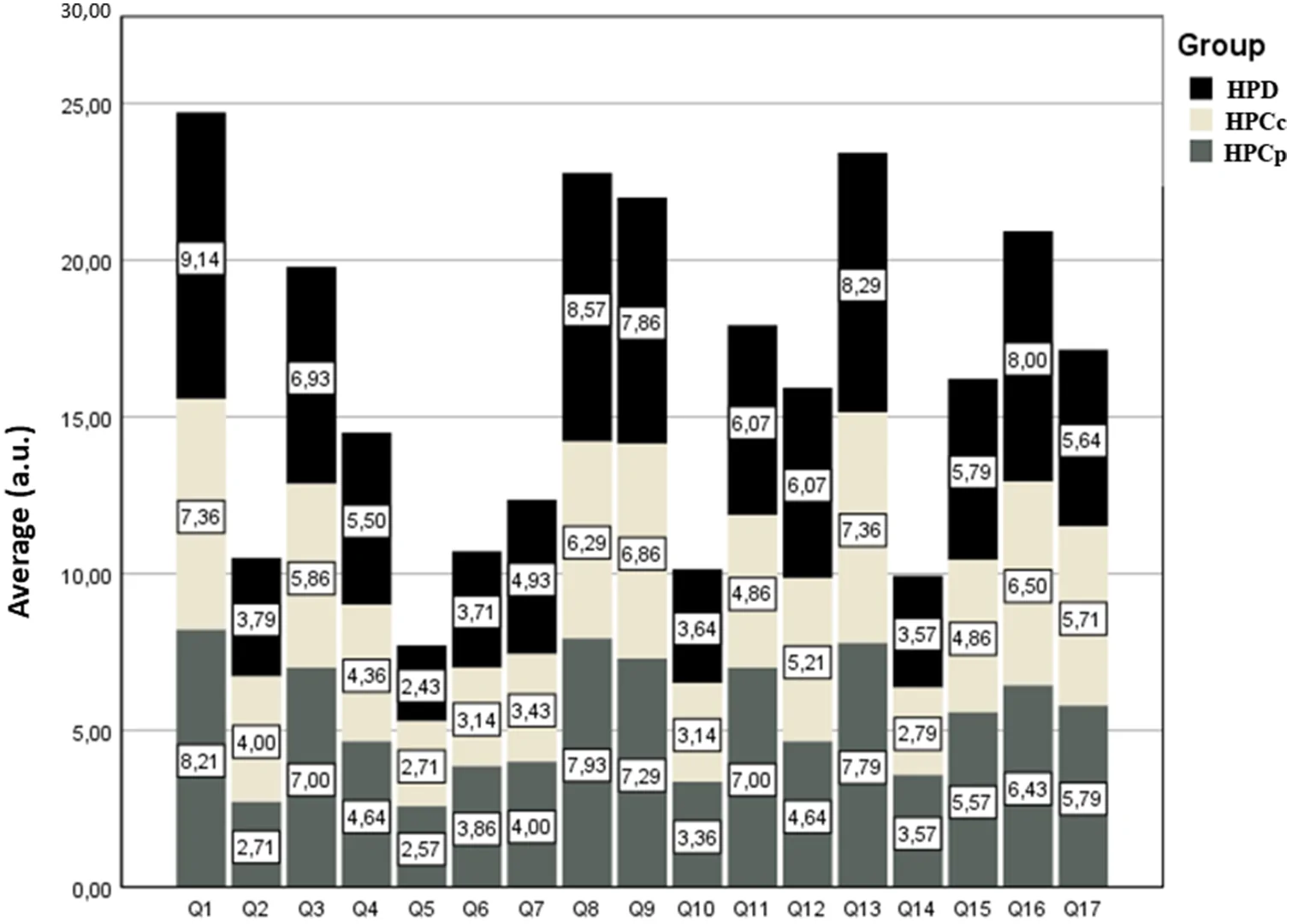
The ANOVA analysis showed that the overall perception between the three groups was significantly different. HPD was distinct from the HPCc (*p* = 0.001) and HPCp responses (*p* = 0.034) r as well HPCc and HPCP were significantly different (*p* = 0.025) a.u. arbitrary units.

A one-way ANOVA revealed significant differences in PERFS scores among the three participant groups [*F_(2, 474)_* *=* *9.510, p* *=* *0.001*]. *Post hoc* analyses indicated that HPDs reported significantly higher ratings than both HPCc (*p* = 0.001) and HPCp (*p* = 0.034). In addition, the responses of HPCc differed significantly from those of HPCp (*p* = 0.025). The overall mean PERFS scores were 5.88 ± 3.00 for HPDs, 4.97 ± 2.76 for HPCc, and 5.43 ± 2.89 for HPCp. Figure 2 illustrates the mean scores for each questionnaire item across participant groups, showing that Items 1, 8, 9, 13, and 16 received the highest overall importance ratings.

Exploratory factor analysis identified five latent factors that together explained 70.88% of the total variance in the questionnaire responses. The first factor accounted for 36.0% of the total variance. The factors were interpreted and labelled according to the conceptual content of the items with the highest loadings as *Process, Technical and Social Support, Structure of Sport Policies, Competition Level, and Contractual Conditions* (Table 2).

**Table 2:** Factors identified as decisive to improve the context of coaches in Colombia and the results in elite sport.

Item	Description	Factors				
		1	2	3	4	5
1	Financial support from the government			X		
2	Financial support from the private company				X	
3	Sports policy and structure of Government/CNO?			X		
4	The massification strategies of your sport	X				
5	The appropriate policies in physical education	X				
6	Talent Detection System	X				
7	Support of sports sciences in an integral way from the level of identification and development of talent	X				
8	Support of sport sciences in an integral way during the high-performance level			X		
9	Access to training scenarios and sports equipment of the highest level			X		
10	Systematic training and certification program for coaches	X				
11	Financial stability and contractual conditions as a coach					X
12	International competition at the best level from the junior category				X	
13	International competition at the best level in the open category				X	
14	Research and scientific innovation applied to sport	X				
15	Social Development area support		X			
16	Sports methodologist support (sport consultant)		X			
17	Support of the administrative secretary of sport		X			
Questions retained by factor		6	3	4	3	1

Factors: 1= Process; 2= Technical & Social support; 3= Structure of sports polices; 4= Competition Level; 5= Contractual conditions.

Regarding the additional comments participants might offer on the factors they considered important for achieving success at the highest levels of international competition, four recurring themes emerged: (1) family support in facilitating athletes' participation in national and international training camps; (2) systematic monitoring of athlete development through the attendance of national coaches at junior and youth championships; (3) the contribution of psychologists and multidisciplinary support teams to the preparation of Paralympic athletes; and (4) the educational background and academic qualifications of high-performance coaches.

## Discussion

This study aimed to identify the factors influencing high-performance sport in a developing country by examining the perceptions of high-performance coaches (HPCs) and high-performance directors (HPDs) in Colombia. The findings are discussed in relation to two complementary dimensions: (1) the strengths and weaknesses of the current coaching environment and (2) the key factors perceived to underpin international sporting success.

### Strengths and weaknesses of the coaching environment

The findings indicate that only four of the twenty support mechanisms assessed in the SUMA questionnaire were perceived as strongly established within the Colombian high-performance sport system. Three of these were directly related to the employment conditions and remuneration of elite coaches, suggesting that coaching at the highest level has achieved a degree of professional recognition within the national sport system. However, this recognition appears to be largely restricted to coaches working with elite athletes.

In contrast, coaches responsible for talent identification and athlete development reported limited employment opportunities, career prospects, and financial support, indicating that the professionalization of coaching remains uneven across different stages of the athlete development pathway.

This imbalance has important implications for the sustainability of elite sport. Although elite coaches may benefit from relatively stable employment, limited investment in coaches working with developing athletes may compromise the long-term quality of talent development. These findings reinforce previous research emphasizing that sustained international success depends on the entire athlete development pathway rather than exclusively on investment at the elite level (36–38).

The study also identified substantial weaknesses in coach education and continuous professional development. Four questionnaire items related to certification, continuing education, and post-career support received the lowest levels of agreement among respondents. These findings suggest the absence of a coherent national coach education framework capable of supporting professional learning throughout a coach's career. Unlike many successful elite sport systems, Colombia currently lacks a national organization responsible for coordinating coach education, certification, and continuing professional development.

Previous research has consistently demonstrated that coaching quality depends not simply on the number of coaches employed within a sport system but on the knowledge, competencies, and learning opportunities available throughout their professional careers. Contemporary coach development increasingly emphasizes reflective practice, contextual learning, and continuous professional development rather than the acquisition of technical knowledge alone (5, 39, 40).

Consequently, strengthening national coach education structures may represent an important opportunity to improve coaching effectiveness and, ultimately, athlete performance.

At the same time, the findings illustrate the distinctive characteristics of coaching in developing countries. Resource limitations often require coaches and athletes to develop adaptive strategies that foster resilience, creativity, and commitment despite structural constraints. Similar observations have been reported by Foster et al. (2022), who argue that challenging environments may stimulate the development of psychological characteristics that contribute to elite performance. Nevertheless, such individual resilience should not be viewed as a substitute for systematic investment in coach development and organizational capacity (41).

Overall, these findings reinforce the importance of considering the broader organizational, governance, and financial context in which coaches operate. Although the support mechanisms evaluated in this study were derived from evidence generated primarily in developed countries, their implementation within developing nations requires consideration of local institutional capacities, governance structures, and available resources.

### Context-independent factors associated with international sporting success

Among the seventeen factors evaluated in the PERFS questionnaire, government financial support, sport science services, training facilities, international competition, and technical advisory support received the highest ratings of importance. These findings closely align with previous studies identifying financial investment, access to high-quality training environments, and regular international competition as fundamental pillars of successful elite sport systems (4, 8, 37, 42, 43).

Although Colombia has progressively expanded social support programs for athletes, including scholarships, accommodation, transportation, nutrition, and psychological services, participants considered these services to remain insufficient. This finding likely reflects the socioeconomic realities experienced by many Colombian athletes, for whom participation in high-performance sport frequently requires overcoming substantial financial and social barriers.

Accordingly, high-performance sport often represents a long-term life project for both athletes and coaches. Achieving international success therefore requires sustained investment not only in elite athletes but also in developing athletes who possess high potential yet lack access to interdisciplinary support services and financial assistance during critical stages of their development.

An additional finding concerns the relatively low importance assigned to national physical education policies. Although this factor received the lowest rating among the seventeen items, the broader literature consistently demonstrates that high-quality physical education contributes to motor competence, lifelong physical activity, and the identification of sporting talent (44–48). Previous reports have shown that Colombian children exhibit relatively low levels of physical activity and that only a small proportion receive physical education delivered by qualified professionals (49, 50). These findings suggest that strengthening school-based physical education may indirectly enhance the long-term development of elite athletes by expanding the national talent pool.

Participants also highlighted the importance of sport science support while simultaneously identifying limited research capacity as an important weakness within the Colombian sport system. Although multidisciplinary services such as sports medicine, physiology, psychology, nutrition, and biomechanics were perceived as valuable contributors to athlete performance, research and innovation received among the lowest ratings in the PERFS questionnaire. This finding suggests that greater integration between researchers and practitioners could strengthen evidence-informed decision-making in talent identification, athlete development, and performance optimization (51–54).

### A framework of performance factors for developing countries

Beyond describing individual support mechanisms, this study identified five broader dimensions underlying perceptions of elite sport success within the Colombian context: Process, Technical and Social Support, Structure of Sport Policies, Competition Level, and Contractual Conditions. Together, these factors provide an empirically derived framework that complements existing models of elite sport development, particularly the SPLISS framework (3, 6, 43) while reflecting the institutional realities of a developing country.

The first dimension, **Process**, which includes organizational capability, emerged as the most influential latent factor. accounting for the largest proportion of variance in participants' responses. Rather than representing a single organizational component, this dimension reflects the capacity of the high-performance sport system to establish coherent processes that support the long-term development of coaches and athletes. The items loading on this factor primarily concerned coach education, certification, continuing professional development, organizational learning, and strategic planning, suggesting that respondents viewed these elements as interconnected components of an effective high-performance system. The consistently low ratings assigned to these items indicate that, despite recent improvements in Colombia's elite sport performance, important structural deficiencies remain in the mechanisms supporting coaches' professional development. In particular, the absence of a nationally coordinated coach education and certification framework limits opportunities for coaches to acquire, update, and transfer knowledge throughout their careers, thereby reducing the system's capacity for continuous improvement. The law regulating the sports coaching profession in Colombia represents a first step toward the important commitment of training and certifying coaches, in line with the systems existing in some developed countries.

These findings are consistent with the broader literature emphasizing that sustainable international sporting success depends not only on financial investment but also on the quality of governance and the organizational processes through which resources are transformed into athlete performance. Within the SPLISS framework, coach development constitutes a central component of the elite sport system and is closely associated with the effectiveness of organizational structures and long-term strategic planning (1, 19). Similarly, governance research has demonstrated that successful sport systems are characterized by capable leadership, coherent decision-making processes, and organizational learning that enables institutions to adapt to changing performance environments (26). From the perspective of coach development, Collins et al. (2016) argue that expertise emerges through continuous reflective practice and context-specific learning rather than through formal qualifications alone. Consequently, strengthening coach education should not be viewed merely as increasing the number of certification programmes but as developing an integrated learning system that combines formal education, mentoring, experiential learning, and evidence-informed practice. Such an approach would enhance the adaptive capacity of the Colombian high-performance sport system and contribute to the long-term sustainability of international sporting success.

The second dimension, **Technical and Social Support**, highlights the importance of multidisciplinary services and athlete support mechanisms in facilitating high-performance outcomes. Participants consistently identified sport science support, technical advisory services, and social assistance programmes as important contributors to international sporting success. These findings suggest that the effectiveness of a high-performance system depends not only on the expertise of coaches but also on the extent to which athletes and coaches are supported by an integrated network of professionals, including sport physicians, physiologists, psychologists, nutritionists, biomechanists, and sport methodologists. In the Colombian context, respondents particularly emphasized the contribution of sport methodologists and multidisciplinary teams to the planning, monitoring, and optimization of training and competition, indicating that technical support has become an increasingly important component of elite sport preparation.

At the same time, participants acknowledged that the availability and quality of social support services remain insufficient to meet the needs of many athletes. Although Colombia has progressively expanded programmes providing scholarships, accommodation, transportation, nutritional assistance, and psychological support for athletes identified as having international medal potential, these initiatives were perceived as only partially addressing the socioeconomic challenges experienced by many high-performance athletes. This finding reinforces the view that athlete development is influenced by broader social and environmental conditions rather than by training alone. Previous research has similarly demonstrated that effective elite sport systems integrate multidisciplinary performance services with comprehensive athlete welfare programmes, recognizing that financial security, educational opportunities, health services, and psychosocial support contribute to both athletic performance and career sustainability (55–57). Consequently, strengthening the integration between technical expertise and social support should be viewed as a strategic investment in the long-term development of athletes and coaches, particularly within resource-constrained sport systems where maximizing the effectiveness of available support services is essential.

The third dimension, **Structure of Sport Policies**, reflects the institutional and governance arrangements that underpin the development of high-performance sport. The items loading on this factor indicate that participants perceived government investment, coherent sport policies, integrated sport science services, and the provision of appropriate training facilities as closely interconnected components of elite sport success. Rather than considering these elements independently, respondents viewed them as part of a coordinated policy environment that supports athlete preparation and international competitiveness. This finding suggests that the effectiveness of elite sport systems depends not only on the amount of resources invested but also on the governance structures through which those resources are planned, coordinated, and implemented.

This interpretation is consistent with previous research demonstrating that sustained international success is associated with coherent policy frameworks, long-term strategic planning, and effective governance rather than financial investment alone (10, 26, 36). Within the SPLISS framework, several policy pillars, including financial support, facilities, sport science services, and governance are considered complementary components of successful elite sport systems. The present findings reinforce this perspective while suggesting that, in the Colombian context, these components are perceived as part of a broader institutional dimension characterized by policy coherence and organizational coordination. Colombia's marked improvement in international performance between 2000 and 2016 appears to illustrate this relationship. During this period, increased public investment was accompanied by the implementation of long-term planning, prioritization of sports and athletes, strengthened technical coordination, and expanded access to multidisciplinary support services. Although subsequent reductions in performance cannot be attributed solely to changes in policy, participants perceived that the weakening of these institutional arrangements coincided with a decline in international competitiveness. This finding highlights the importance of policy continuity and stable governance in sustaining elite sport performance over time.

The fourth dimension, **Competition Level**, underscores the importance of regular participation in high-level international competition as a critical component of elite athlete development. Participants consistently identified participation in international competitions as one of the most influential factors contributing to successful performances at the Olympic Games, World Championships, and other major international events. This finding supports the view that competitive success is developed progressively through repeated exposure to increasingly demanding performance environments, where athletes acquire not only technical and tactical competencies but also the psychological skills required to perform under the pressures of international competition.

Within the SPLISS framework, competitive opportunities constitute an essential mechanism through which athlete development pathways are strengthened and international competitiveness is enhanced. Importantly, participants in the present study emphasized that expanding international competition opportunities should not be limited to senior elite athletes but should also include junior and developing athletes. This observation highlights the importance of constructing a continuous competitive pathway that enables talented athletes to accumulate international experience before reaching senior competition. Such an approach is particularly relevant for developing countries, where financial constraints frequently limit participation in international events and may reduce athletes' opportunities to acquire the competitive experience necessary to successfully transition to the highest levels of international sport. Consequently, investment in international competition should be viewed not merely as support for current elite athletes but as a strategic component of long-term talent development and sustained international performance (13, 37, 58, 59).

Finally, **Contractual Conditions** highlights the importance of employment stability and professional recognition in strengthening high-performance sport systems. Participants perceived competitive salaries, long-term contracts, career security, and opportunities for professional advancement as interconnected elements that influence coaches' ability to perform effectively and remain within the elite sport system. Unlike many other factors identified in this study, contractual conditions were viewed not simply as administrative arrangements but as strategic mechanisms for attracting, developing, and retaining highly qualified coaches. These findings suggest that the long-term success of elite sport systems depends not only on recruiting talented coaches but also on creating employment conditions that allow them to invest in long-term athlete development without the uncertainty associated with short-term or unstable contracts.

This interpretation is consistent with the growing recognition that coaches represent one of the most valuable human resources within elite sport systems. The SPLISS framework identifies coach provision and development as a key policy pillar, emphasizing that investments in coach expertise are essential for sustained international success (43). Likewise, research on sport governance and coach development argues that effective high-performance systems require employment structures that promote continuity, professional autonomy, and organizational commitment, enabling coaches to engage in long-term planning and continuous learning rather than focusing on short-term performance outcomes (5, 40).

In the Colombian context, participants perceived that improving contractual conditions would contribute not only to retaining experienced coaches but also to attracting highly qualified national and international professionals capable of strengthening the country's coaching capacity. Consequently, policies aimed at enhancing contractual stability and professional recognition should be viewed as strategic investments in the sustainability and competitiveness of the high-performance sport system rather than as personnel management issues alone.

In the other hand, although direct employment by the Ministry of Sport and the Colombian Olympic Committee represents an important form of professional recognition, participants expressed concerns regarding the short-term nature of employment contracts and interruptions between contract periods. Such employment instability may negatively affect continuity in athlete preparation and reduce the capacity to attract and retain highly qualified national and international coaching personnel.

Taken together, these findings suggest that the determinants of elite sport success extend beyond financial investment alone. Organizational capacity, governance quality, professional development, interdisciplinary support, and stable employment conditions collectively shape the environment within which athletes and coaches pursue international success. Consistent with previous studies the present findings further suggest that understanding elite sport performance requires identifying the context-specific combination of factors that facilitate or constrain success within individual national sport systems from the perspective of HPC and HPD (13, 26, 55, 60, 61).

### Limitations

Several limitations should be considered when interpreting the findings of this study. First, the sample was restricted to high-performance coaches and high-performance directors working within the Colombian elite sport system. Consequently, the five-dimensional framework identified in this study reflects the perceptions of stakeholders operating at the highest levels of sport and should not be generalized to coaches working in other contexts, such as community sport, school sport, youth development programs, or private sport organizations.

In addition, the sample was primarily composed of coaches from individual sports. As the organizational structures, resource requirements, and coaching practices of team sports may differ substantially, future research should examine whether the factor structure identified in this study is applicable across different sporting contexts and disciplines.

Second, the exploratory factor analysis was conducted using a relatively small, purposefully selected sample drawn from a finite population of national high-performance coaches and directors. However, it should be remembered that this approach was appropriate for the exploratory objectives of the study and represents a substantial proportion of the target population, the resulting factor structure should be interpreted as preliminary. Future studies involving larger samples from multiple countries should examine the stability and generalizability of the proposed framework using confirmatory analytical approaches.

Finally, the analysis of the SUMA questionnaire focused primarily on participants' dichotomous (Yes/No) responses, while the qualitative comments accompanying these responses were used only to support the interpretation of the quantitative findings. These narrative data may contain valuable contextual information regarding the organizational, cultural, and policy-related factors influencing elite sport development in developing countries. Future research could employ qualitative or mixed-methods approaches to explore these perspectives in greater depth and further refine the dimensions identified in the present study.

Despite these limitations, this study provides one of the first empirically derived frameworks describing the organizational dimensions perceived to influence elite sport development in a South American context.

## Conclusion

Although existing frameworks of elite sport success, such as SPLISS, provide valuable insights into the determinants of international sporting performance, their application to developing countries requires careful consideration of the institutional, organizational, and financial contexts in which elite sport systems operate. The findings of this study demonstrate that stakeholders within the Colombian high-performance sport system perceive the relative importance and interaction of these determinants differently, with high-performance directors generally reporting more favourable evaluations than high-performance coaches. These differences highlight the value of incorporating multiple stakeholder perspectives when assessing the effectiveness of elite sport policies and programmes.

More importantly, this study identified five interrelated dimensions that characterize the Colombian high-performance sport environment: Process, Technical and Social Support, Structure of Sport Policies, Competition Level, and Contractual Conditions of Coaches. Rather than functioning as isolated determinants of success, these dimensions represent an integrated framework through which organizational capacity, governance, technical expertise, competitive opportunities, and human resource management collectively shape elite sport development. The findings therefore suggest that improving high-performance sport systems requires greater attention not only to the presence of specific policy components but also to the quality of their implementation, coordination, and interaction within the broader sport system. In this respect, elite sport success depends not simply on possessing the appropriate resources, but on how effectively these resources are organized, integrated, and strategically managed.

By examining elite sport development through the perspectives of high-performance coaches and high-performance directors, this study contributes to the growing literature on elite sport policy in developing countries and provides an empirically derived framework that may inform both future research and policy development. Future studies should examine the applicability of this five-dimensional framework across different sports and national contexts and assess its stability using larger samples and confirmatory analytical approaches. Such research would contribute to a more comprehensive understanding of how elite sport systems can be designed and managed to achieve sustainable international sporting success across diverse institutional settings.

## Data Availability

The raw data supporting the conclusions of this article will be made available by the authors, without undue reservation.
