# Supplementary material for: High-performance sport in a developing country through the lens of coaches and sports directors
Source: Front Sports Act Living. 2026 Sep 3;8:1877793. doi: 10.3389/fspor.2026.1877793 (PMC13582573; doi:10.3389/fspor.2026.1877793)
Supplement: Supplementary file 1 [file Supplementaryfile1.docx]

Questionnaire 1: SUMA (Support mechanisms available).
Objective: To evaluate the context and development of high performance coaches (Talent development and Elite athletes).

Note: mark with an "X" in the cell as appropriate

No.	ITEM	YES	NO	I Don't know	COMMENTARY (Optional)
1	There is a database of coaches and elite coaches that is updated yearly, and contains details of qualifications and the date qualifications were achieved.				
2	Coaches have experience at the elite level in their own career as an athlete.				
3	The Ministry os sport or the Public Institute of the highest hierarchy in your country and / or the COC have a strategy for NGBs to attract the world's best coaches and external experts to train elite athletes and to improve the expertise of domestic coaches working at elite level.				
4	There is a nationally coordinating agency (or Into Coldeportes) responsible for coaches' education in general and elite coach education in particular.				
5	If this organization exists, she aligns with the different levels of NGB courses and facilitates NGBs in the organisation of coach development and defines coaching profiles.				
6	There is a well developed coach education system from the lowest level (courses for the recreational trainer / coach) to the highest level (education of elite coaches).				
7	There are several services (such as regular refresher courses, information exchange opportunities) and resources supporting the continuous professional development of coaches.				
8	Coaches can receive specialist advice from other areas to help them improve the standard of their athletes (psychology, nutrition, physiology, biomechanics, data analysis).				
9	Coaches' general monthly income (total gross annual income) plus income from their sport activities is sufficiently high to provide a good standard of living.				
10	Elite sport coaching is –or can be- a full time primary activity for the best elite coaches.				
11	Employers are supportive by taking into account the training needs of elite coaches.				
12	The job of a coach is recognised in the country and the career prospects are high.				
13	Coaches have a written work contract for training activities; the job of a coach is contractually recognized and protected.				
14	If there is a contract, it is renewed annually, every two years, every 4 years, indefinitely.				
15	There is a trade union for sports coaches and trainers.				
16	Elite coaches receive a post career support programme to prepare and assist for life after sport.				
17	A coaching qualification is mandatory to work in sport clubs and with young talents.				
18	Can coaches working with potential young athletes (talent development) receive direct financial support that is sufficient for them to work as full-time coaches.				
19	Coaches earn bonuses ($) related to the performance of their athletes.				
20	Coaches are allowed to earn additional money (for example, through sponsorships) in addition to the payment they receive for their sports organization.				
		0	0	0	
