# Supplementary material for: High-performance sport in a developing country through the lens of coaches and sports directors
Source: Front Sports Act Living. 2026 Sep 3;8:1877793. doi: 10.3389/fspor.2026.1877793 (PMC13582573; doi:10.3389/fspor.2026.1877793)
Supplement: Supplementary file 2 [file Supplementaryfile2.docx]

Questionnaire 2: PESF (Perception of success factors)

Objective: to establish the priority level of the listed items that, according to you, have allowed your athletes to achieve results at the highest competitive level (World Championships, Olympic Games).

Note: mark with an "X" in the cell as appropriate

No.	ITEM	1	2	3	4	5	6	7	8	9	10	COMMENTARY (Optional)
1	Financial support from the government											
2	Financial support from the private company											
3	Sports policy and structure of Government / CNO?											
4	The massification strategies of your sport											
5	The appropriate policies in physical education											
6	Talent Detection System											
7	Support of sports sciences in an integral way from the level of identification and development of talent											
8	Support of sport sciences in an integral way during the high performance level											
9	Access to training scenarios and sports equipment of the highest level											
10	Systematic training and certification program for coaches											
11	Financial stability and contractual conditions as a coach											
12	International competition at the best level from the junior category											
13	International competition at the best level in the open category											
14	Research and scientific innovation applied to sport											
15	Social Development area support											
16	Sports methodologist support(sport consultant)											
17	Support of the administrative secretary of sport											
		0	0	0	0	0	0	0	0	0	0	
